# Universal antibiotic tolerance arising from antibiotic-triggered accumulation of pyocyanin in Pseudomonas aeruginosa

**DOI:** 10.1371/journal.pbio.3000573

**Published:** 2019-12-16

**Authors:** Kui Zhu, Shang Chen, Tatyana A. Sysoeva, Lingchong You

**Affiliations:** 1 Beijing Advanced Innovation Center for Food Nutrition and Human Health, College of Veterinary Medicine, China Agricultural University, Haidian, Beijing, China; 2 Department of Biomedical Engineering, Duke University, Durham, North Carolina, United States of America; 3 Center for Genomic and Computational Biology, Duke University, Durham, North Carolina, United States of America; 4 Department of Molecular Genetics and Microbiology, Duke University School of Medicine, Durham, North Carolina, United States of America; Hebrew University, ISRAEL

## Abstract

*Pseudomonas aeruginosa* is an opportunistic pathogen that often infects open wounds or patients with cystic fibrosis. Once established, *P*. *aeruginosa* infections are notoriously difficult to eradicate. This difficulty is in part due to the ability of *P*. *aeruginosa* to tolerate antibiotic treatment at the individual-cell level or through collective behaviors. Here, we describe a new phenomenon by which *P*. *aeruginosa* tolerates antibiotic treatment. In particular, treatment of *P*. *aeruginosa* with sublethal concentrations of antibiotics covering all major classes promoted accumulation of the redox-sensitive phenazine pyocyanin (PYO). PYO in turn conferred general tolerance against diverse antibiotics for both *P*. *aeruginosa* and other gram-negative and gram-positive bacteria. This property is shared by other redox-active phenazines produced by *P*. *aeruginosa*. Our discovery sheds new insights into the physiological functions of phenazines and has implications for designing effective antibiotic treatment protocols.

## Abstract 2

Treatment of the pathogenic bacterium Pseudomonas aeruginosa with sublethal concentrations of diverse antibiotics promotes the accumulation of pyocyanin, an important virulence factor; in turn, pyocyanin confers general tolerance against antibiotics for both P. aeruginosa and other bacteria.

## Introduction

The overuse and misuse of antibiotics has led to a global crisis [1]: bacteria have developed resistance against every existing antibiotic and are doing so at an alarming rate, considering the timescale at which new antibiotics progress from development to clinical application [2,3]. The drying antibiotic pipeline further heightens the global threat created by infectious bacteria [4,5]. A critical approach to the problem is developing ways to revitalize existing antibiotics [6]. To extend the use of existing antibiotics, we need to develop a mechanistic understanding of the diverse ways by which bacteria survive antibiotics. Such an understanding is critical for designing therapeutic approaches that subvert the survival tactics by bacterial pathogens.

Past efforts on antibiotic resistance have focused on responses of individual cells, such as mutations in the antibiotic targets, enzymatic activity that inactivates antibiotics, and increased activation of efflux pumps [6]. Unlike antibiotic resistance, which is due to inherited or acquired mutations [7,8], tolerance reflects the ability of individual cells [9] or cell populations [10] to survive antibiotic treatment without acquiring new mutations. However, it has been long realized that antibiotic tolerance precedes resistance [11–13]. Recent in vitro experiments have shown that antibiotic tolerance can facilitate the evolution of resistance [14]. In particular, antibiotic tolerance can pave the way for the rapid subsequent emergence of antibiotic resistance in bacteria; therefore, preventing the evolution of tolerance may shed light on alternative strategies for antibiotic treatments. Importantly, the ability to survive antibiotic treatment is typically considered an intrinsic property of the single bacterial cells or bacterial populations, before antibiotics are applied. In contrast to these prevailing views, here we describe a previously unknown phenomenon of collective antibiotic tolerance in *Pseudomonas aeruginosa*, whereby bacterial growth under subinhibitory concentrations of antibiotics is promoted by certain phenazines.

Pyocyanin (PYO), one of the most studied phenazines, is a redox-active metabolite giving the characteristic blue-green pigment of *P*. *aeruginosa* cultures [15]. PYO is typically produced when *P*. *aeruginosa* enters the stationary phase when the cell density is high [16]. Recent studies have demonstrated a number of physiological roles of PYO, such as serving as a signaling compound [17], facilitating biofilm development [18], promoting iron acquisition [19], and influencing colony formation [20]. It is also known to confer a broad-spectrum antibiotic activity against other microorganisms [21]. Complementary to these studies, our work reveals a new role of PYO and other phenazines in promoting the survival of bacteria during antibiotic treatment.

## Results

### Subinhibitory concentrations of antibiotics induce PYO accumulation

**Fig 1:**
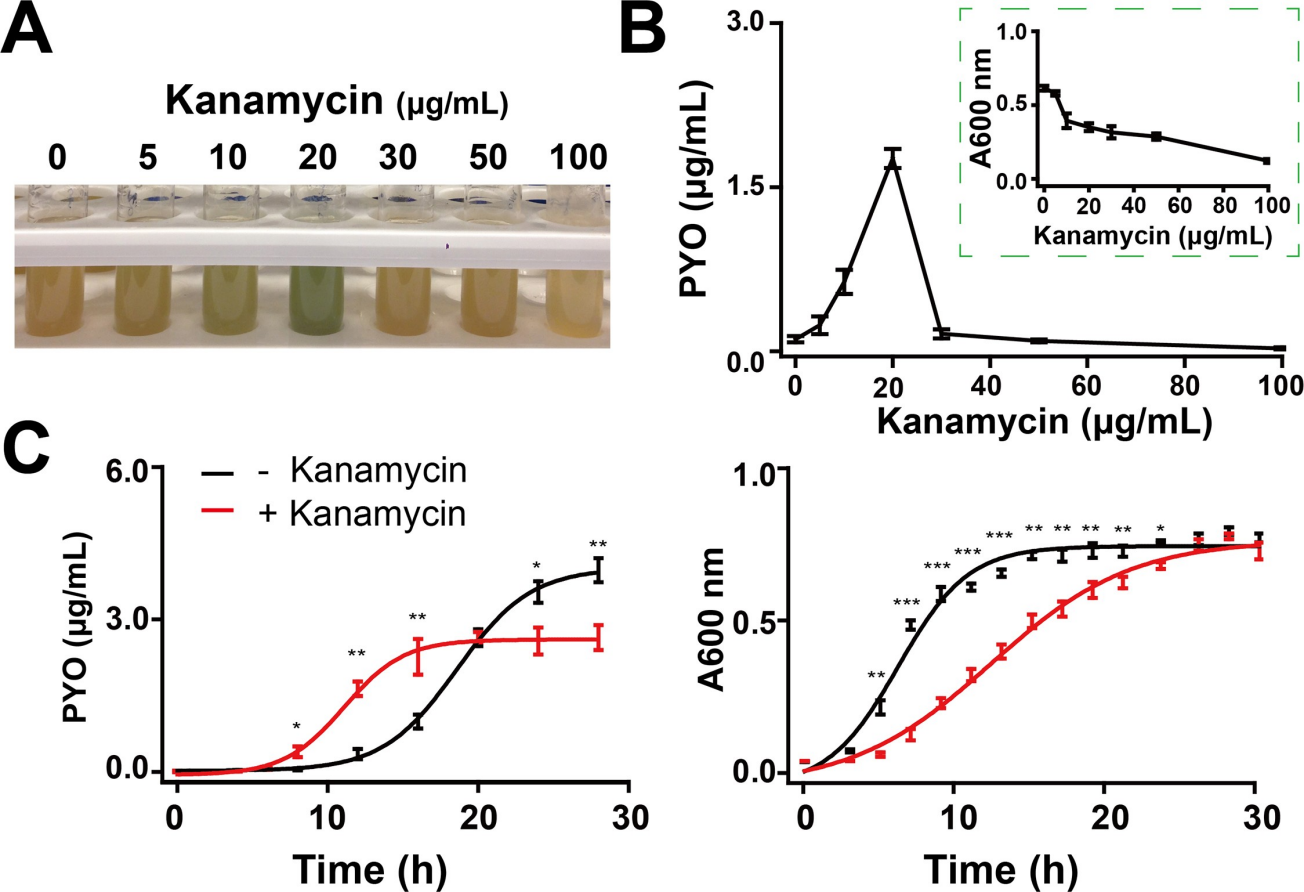
Antibiotics induced accumulation of PYO. (A) Representative images of Kan-induced color changes in *P*. *aeruginosa* PAO1 cultured in LB medium, with shaking of 200 r/min at 37°C. The image of the tubes was taken at 12 h. (B) PYO concentrations and cell densities (A600 nm) of PAO1 in varying concentrations of Kan after incubation in the tubes for 12 h. Regarding A and B, bacteria in the presence and absence of Kan are in the exponential phase and early stationary phase, respectively. (C) Typical dynamics of PYO concentrations (left) and bacterial growth curves (right) of PAO1 treated with 20 μg/mL Kan in the tubes, with shaking of 200 r/min at 37°C. The data underlying this figure can be found in S1 Data. Means ± SD are presented (*n* = 3). *P < 0.05, **P < 0.01, ***P < 0.001. P values were determined by paired t test. A600 nm, absorbance 600 nm; Kan, kanamycin; LB, Luria-Bertani; PYO, pyocyanin.

We first observed that subinhibitory concentrations of kanamycin (Kan), a commonly used aminoglycoside antibiotic, induced a blue-greenish color change in *P*. *aeruginosa* PAO1 (American Type Culture Collection [ATCC] 47085) cultures. PYO is a typical pigment for such color change [15]. We confirmed this notion by purifying PYO using an established method [22], which also allowed us to measure PYO accumulation. The induction of PYO followed a biphasic dependence on the Kan dose. The blue-green color of the cultures became more visible with increasing Kan concentrations (Fig 1A). This color change is consistent with direct quantification of PYO in the supernatants (Fig 1B). Concurrently, the bacterial density (absorbance 600 [A600] nm) decreased with increasing Kan concentrations (Fig 1B). As shown in Fig 1C, the levels of PYO in the Kan-treated culture was higher than the control, from 10 h to 16 h. Subsequently, the trend was reversed.

We wondered whether the enhanced PYO accumulation could represent a general stress response to antibiotic treatment. To test this notion, we measured PAO1 strain responses to other antibiotics with different modes of action, including chloramphenicol, which targets the 50S subunit of ribosome, norfloxacin (quinolone family), which inhibits DNA replication, polymyxin B (polypeptide family), which alters cell-membrane permeability, and carbenicillin (β-lactams), which inhibits cell-wall synthesis. All tested antibiotics promoted accumulation of PYO in a similar biphasic manner (S1A Fig). This response was not unique to PAO1. The PA14 strain, which is known to produce more PYO than PAO1 [17], exhibited qualitatively the same responses to these antibiotics (S1B Fig). Additionally, similar color changes were also observed in clinical isolates in artificial sputum medium (S2 Fig). The biphasic PYO accumulation and its temporal dynamics reconcile the apparently contradictory conclusions on PYO accumulation under antibiotic stresses measured previously by single-point measurements [23–25]. Further studies are needed to elucidate to what extent—and how—sublethal levels of antibiotics modulate the accumulation of PYO.

### Accumulated PYO enhances antibiotic tolerance in bacteria

**Fig 2:**
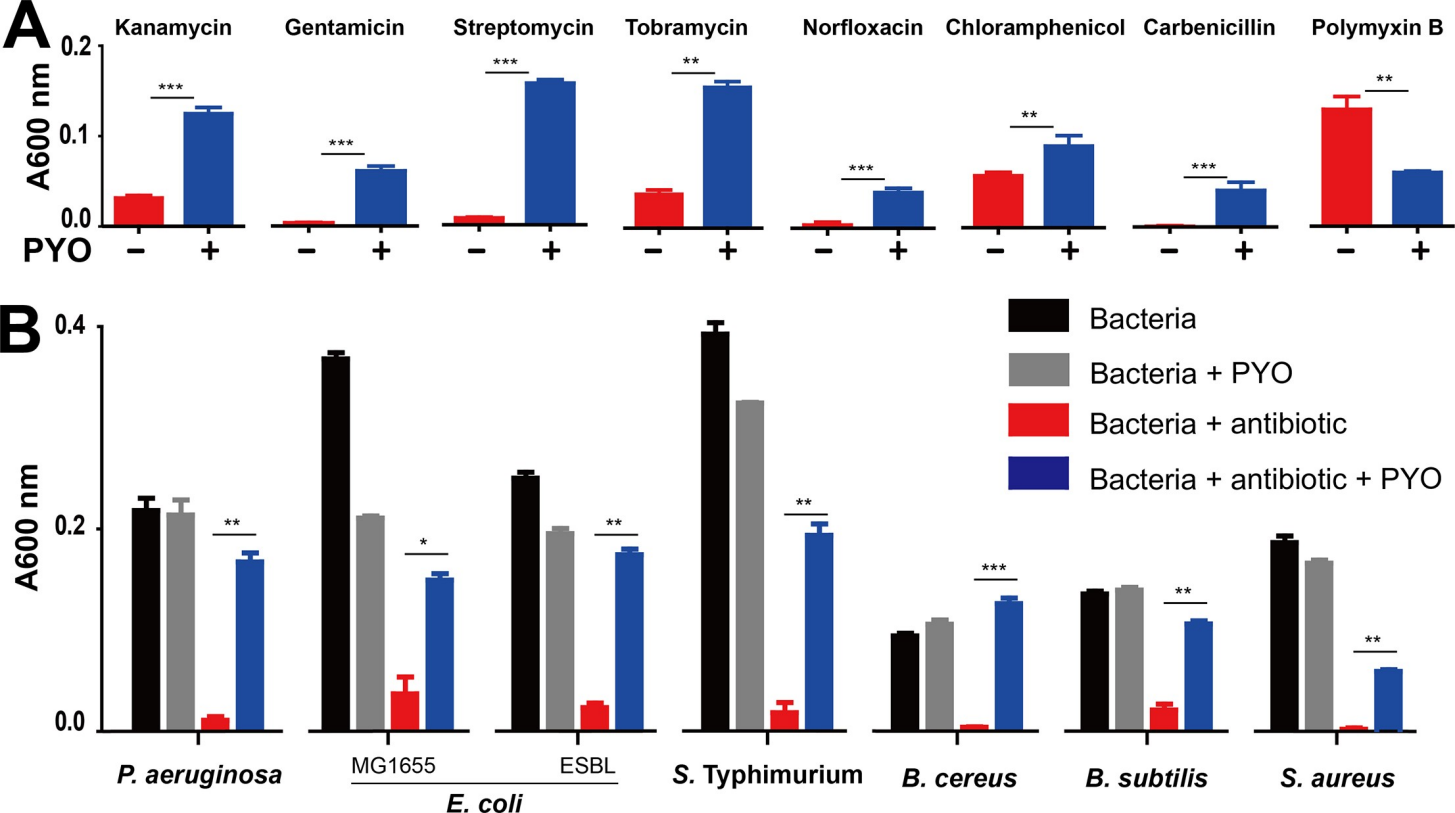
PYO-mediated tolerance against antibiotic treatments. (A) PYO-mediated tolerance in *P*. *aeruginosa* PAO1. PAO1 in the presence of 2 μg/mL PYO was treated with various antibiotics, including 50 μg/mL Kan, 4 μg/mL gentamicin, 20 μg/mL streptomycin, 20 μg/mL tobramycin, 0.5 μg/mL norfloxacin, 7.5 μg/mL chloramphenicol, 50 μg/mL carbenicillin, and 1.0 μg/mL polymyxin B. All data were plotted from the growth curves of various bacterial species at 10 h. Means ± SD are presented (*n* = 3). (B) PYO-mediated tolerance in gram-negative bacteria, including *P*. *aeruginosa* PAO1, *Escherichia coli* (MG1655- and ESBL-expressing strain), and *Salmonella typhimurium*, and gram-positive bacteria, including *Bacillus cereus*, *B*. *subtilis*, and *Staphylococcus aureus*. Bacteria were cultured in LB media supplemented with 20 μg/mL streptomycin, except 5 μg/mL streptomycin for *B*. *cereus*. Means ± SD are presented (*n* = 6). The data underlying this figure can be found in S1 Data. *P < 0.05, **P < 0.01, ***P < 0.001; *P* values were determined by paired *t* test. A600 nm, absorbance 600 nm; ESBL, extended-spectrum beta-lactamase; Kan, kanamycin; LB, Luria-Bertani; PYO, pyocyanin.

We wondered whether the enhanced accumulation of PYO could represent a survival mechanism for the population under acute antibiotic stress. Indeed, growth of *P*. *aeruginosa* PAO1 in the presence of Kan was enhanced by exogenously added PYO (Fig 2A). This PYO-mediated tolerance was effective against other aminoglycosides (gentamicin, streptomycin, and tobramycin) and antibiotics of other classes (norfloxacin, chloramphenicol, and carbenicillin) (Fig 2A). In the presence of PYO, PAO1 cultures exhibited a much shorter lag time before recovering in the presence of an antibiotic, in comparison to cultures without exogenously added PYO (S3 Fig). We speculate that the endogenous PYO produced by *P*. *aeruginosa*, if accumulated at a sufficiently high level such as in the cystic fibrosis respiratory tract [26] or at stationary phase [17], could also provide protection. In addition, PYO-mediated tolerance was maintained when PAO1 was cultured in other media or different oxygen conditions (S4 Fig). An exception to the general PYO-mediated tolerance was polymyxin B, for which PYO enhanced ability to inhibit bacterial growth (Fig 2A).

In addition to PAO1, PYO conferred antibiotic tolerance to several other strains of *P*. *aeruginosa*, including PA1C (ATCC 15692), PAO-JP2 with *ΔlasI* and *ΔrhlI* [27], PAO1-W and its mutants (PAO-mxM and PAO-mxS) [28], and PA14 and its mutant (PA14 *Δphz*) [17]. Among these, PAO-mxM, PAO-mxS, and PA14 *Δphz* cannot synthesize PYO, indicating that PYO-mediated tolerance is independent of the strain’s ability to synthesize it (S5 Fig). Furthermore, the growth curves of PA14 and PA14 *Δphz* showed that exogenous PYO alleviated the suppression under antibiotic treatment (S6 Fig). These results suggest that, though wild-type *P*. *aeruginosa* is able to produce PYO endogenously, the endogenous PYO produced by a low-density culture was too low to offer sufficient protection. Given the fact that PYO can accumulate to more than 10 μg/mL in a high-density culture [17], such a concentration of PYO could be sufficient to rescue an otherwise sensitive population. To test this notion, we examined whether the supernatants of high-density cultures of PA14 and PA14 *Δphz* could offer protection for PAO1 under antibiotic treatment (S7 Fig). The supernatant of PA14 *Δphz* did not provide any protection for PAO1 in the presence of antibiotic, but that of PA14 did.

We note that the tolerance was maintained for other gram-negative (*E*. *coli*, *S*. *typhimurium*) and gram-positive (*B*. *cereus*, *B*. *subtilis*, and *S*. *aureus*) bacteria treated with antibiotics (Fig 2B). Futher studies are needed to elucidate how phenazines—particularly PYO—potentiate survival of bacteria under antibiotic treatments and what the biological significance is.

## Discussion

Subinhibitory concentrations of antibiotics can trigger a diverse range of bacterial processes [29]. The antibiotic-induced early PYO accumulation may result from increased production of PYO because bacteria could increase PYO synthesis to counteract stress, akin to that experienced by bacteria entering the stationary phase. It has been well established that different antibiotics can trigger accumulation of reactive oxygen species (ROS) [30]. Likewise, quorum sensing (QS) has been implicated in PYO metabolism [17]. Nevertheless, how bacteria modulate the accumulation and its biological significance remains unknown.

Our results reveal a previously unknown phenomenon whereby *P*. *aeruginosa* can develop collective antibiotic tolerance [10] mediated by PYO. Both the induction of PYO and the PYO-mediated tolerance are general: all tested antibiotics promoted accumulation of PYO; PYO conferred tolerance against all antibiotics with the exception of polymyxin B, which targets bacterial membrane (Fig 2A). Moreover, the PYO-mediated protection is not limited to the producing cells; rather, PYO also enhances survival of other *P*. *aeruginosa* strains and other bacteria. This property highlights a particular challenge in treating infections involving *P*. *aeruginosa*: incomplete suppression of *P*. *aeruginosa* by antibiotics would enhance growth of survivors, as well as other pathogens in the growth environment. Thus, this tolerance also presents an opportunity for designing treatment strategies that can synergistically enhance effects of antibiotics. For instance, specific inhibitors as adjuvants can be developed to target the multiple enzymes involved for the synthesis of phenazines, to counteract the protective effect of PYO.

## Materials and methods

### Chemicals and strains

Antibiotics used in this study include carbenicillin, chloramphenicol, gentamicin, Kan, norfloxacin, polymyxin B, streptomycin, and tobramycin. Oxidized forms of PYO and other phenazines were prepared as previously described [1]. All chemicals were purchased from Sigma-Aldrich and Cayman Chemical. *P*. *aeruginosa*, *E*. *coli*, *S*. *typhimurium*, *B*. *cereus*, *B*. *subtilis*, and *S*. *aureus* strains were used in this study. More details of these strains are shown in S1 Table.

### PYO accumulation in culture tubes

Different concentrations of antibiotics were added to 4 mL Luria-Bertani (LB) broth (5 g/L yeast extract, 10 g/L tryptone, and 10 g/L NaCl, Genesee Scientific) in culture tubes (Genesee Scientific) for *P*. *aeruginosa* PAO1 and PA14 strains. Cultures at exponential phase were adjusted to A600 nm = 0.2; subsequently, 10 μL cultures were resuspended in 4 mL fresh LB medium. All the tubes were cultured in a shaker (New Brunswick Scientific) at 37°C, 250 rpm. When appropriate, the photos of cultured tubes treated with various antibiotics were taken, and cell density (A600 nm) was measured at 600 nm. Excreted PYO was quantified based on the presence of pink to deep red color in acidic solution as described previously [2]. Briefly, 2.5 mL supernatant from LB broth was mixed with 1.5 mL chloroform at specified time points. PYO in the chloroform phase was then extracted into 0.5 mL 0.2 mol/L hydrochloric acid (HCl). After centrifugation, the absorbance of the top layer was measured at 520 nm. Concentrations (micrograms per milliliter) of PYO were expressed as micrograms of PYO per milliliter supernatant, determined by multiplying the absorbance at 520 nm (A520 nm) by 17.072.

### Tolerance experiments

For routine growth, bacterial strains were all cultured in 100 μL LB medium in 96-well cell culture plates (Costar, Corning) for long-term measurement with a plate reader (Victor, Perkin Elmer). Exponential growth cultures of bacteria were adjusted to A600 nm = 0.2, then 1:20 diluted in 100 μL fresh LB broth containing various concentrations of antibiotics either in the presence or absence of exogenous 2 μg/mL PYO or other phenazines. *P*. *aeruginosa* PAO1 was also cultured in other media, including brain-heart infusion (BHI), M9, and 2xYT media (BD Difco). For growth dynamic measurements, 50 μL mineral oil (Sigma) was added into each well to prevent evaporation. The absorbance was measured by an Infinite 200 Pro plate reader (Tecan) at the wavelength of 600 nm (A600). Furthermore, an oxygen-permeable sealing membrane (Diversified Biotech) was used to determine whether increasing oxygen concentration could increase antibiotic tolerance.

### Statistical analysis and software

The statistical significance of differences was determined by a paired *t* test. The fluorescent intensities and fold changes in mRNA levels were normalized prior to analysis. GraphPad Prism 5.0 was used for analyzing the data and generating figures.

## Supporting information

S1 Table Bacterial strains used in this study. (XLSX)

S1 Fig Antibiotics induced accumulation of PYO in *P*. *aeruginosa*. Antibiotics induced color changes in *P*. *aeruginosa* PAO1 (A) and PA14 (B) cultured in LB. Five kinds of antibiotics, including streptomycin/Kan, chloramphenicol, norfloxacin, polymyxin B, and carbenicillin, were tested by endpoint assays. The photos were taken at different time points for different antibiotics to best illustrate the color change in the cultures. The MICs of streptomycin/Kan, chloramphenicol, norfloxacin, polymyxin B, and carbenicillin for *P*. *aeruginosa* PAO1 (A) and PA14 were 8/32, 32, 0.8, 0.8, and 64 μg/mL and 16/64, 64, 0.25, 0.4, and 64 μg/mL, respectively. MICs were determined by micro-broth dilution method according to the CLSI’s operating directions (CLSI M100S-S26). CLSI, Clinical and Laboratory Standards Institute; MIC, minimum inhibitory concentration.
(TIF)

S2 Fig Representative images of sub-MIC antibiotics induced color changes in clinical isolates and PA14 in LB and ASM. (A) *P*. *aeruginosa* 1011 was treated with norfloxacin in LB for 16 h. *P*. *aeruginosa* 1011 was treated with norfloxacin in ASM for 20 h. (B) PA14 was treated with norfloxacin in ASM for 20 h. (C) *P*. *aeruginosa* 1613 was treated with gentamicin in ASM for 24 h. (D) The clinical *P*. *aeruginosa* isolates (strains 1011 and 1613) were kindly gifted by Professor Rong Zhang at The Second Affiliated Hospital of Zhejiang University (Hangzhou, China). ASM was prepared according to the protocol shared by Sriramulu Diraviam Dinesh (Protocol Exchange, 2010; doi:10.1038/protex.2010.212). All of the *P*. *aeruginosa* were cultured in tubes with shaking of 200 r/min at 37°C. The data underlying this figure can be found in S1 Data. ASM, artificial sputum medium; MIC, minimum inhibitory concentration.
(TIF)

S3 Fig PYO-mediated tolerance to various antibiotics. (A) The tolerance was PYO-dose dependent. PAO1 was treated with 20 μg/mL Strep and 30 μg/mL Strep. Culture of *P*. *aeruginosa* PAO1 at exponential phase (A600 nm = 0.2) was diluted in LB broth, then treated with different antibiotics, in the presence (blue line) or absence (red line) of 2 μg/mL PYO. (B) Growth curves of PAO1 treated with varying concentrations of tobramycin (from 10 μg/mL to 50 μg/mL, as indicated), in the presence (blue line) or absence (red line) of 2 μg/mL PYO. (C) Growth curves of PAO1 treated with polymycin B (at 1 μg/mL and 2 μg/mL), in the presence (blue line) or absence (red line) of 2 μg/mL PYO. A600 nm was measured by a plate reader. Means ± SD are presented throughout (*n* = 3). The data underlying this figure can be found in S1 Data. Strep, streptomycin.
(TIF)

S4 Fig PYO-mediated tolerance in various nutrient contents and oxygen conditions. (A) PYO-mediated tolerance in LB broth (A-1), BHI (A-2), M9 (A-3), and 2xYT (A-4) media. Cultures of *P*. *aeruginosa* PAO1 were inoculated into the media in the presence of 20 μg/mL streptomycin and 2 μg/mL PYO. The growth curves were measured by A600 nm in a 96-well plate with a plater reader. Means ± SD were presented throughout (*n* = 3). (B) PYO-mediated tolerance in both oxygen-rich and oxygen-poor environments. *P*. *aeruginosa* PAO1 reached higher densities in an oxygen-rich condition (wells were sealed with oxygen-permeable membrane) than in an oxygen-poor condition (wells were covered with mineral oil). The ratios are 0.749 and 0.650 under oxygen-rich and oxygen-poor conditions, respectively. Means ± SD were presented throughout (*n* = 6). The data underlying this figure can be found in S1 Data.
(TIF)

S5 Fig PYO-mediated tolerance in various *P*. *aeruginosa* strains. (A) PYO-mediated tolerance in *P*. *aeruginosa* PAO1 and mutants. PAO1 (A-1), PA1C (A-2), PAO-JP2 (A-3), PAO1-W, and mutants (A-4 to A-6) were treated with 20 μg/mL, 30 μg/mL, 20 μg/mL, and 30 μg/mL streptomycin, respectively, in the presence or absence of 2 μg/mL PYO. (B) PYO-mediated tolerance was independent of the endogenous PYO production. PA14 (B-1, wild type), PA14 *Δphz* (B-2, no PYO production), and DKN370 (b-3, PYO-overproducing strain) were treated with 20 μg/mL streptomycin in the presence or absence of 2 μg/mL PYO. All data were plotted at 10 h after inoculation. Means ± SD are presented throughout (*n* = 6). This apparent independence was likely due to the lack of sufficient PYO accumulation at an initially low-density culture, even if a strain has the ability to produce PYO. Also see results in S6 and S7 Figs. The data underlying this figure can be found in S1 Data.
(TIF)

S6 Fig PYO-mediated antibiotic tolerance in PA14 and PA14 *Δphz*. (A) Growth curves of PA14 (WT) without treatment or treated with 20 μg/mL Strep, 2 μg/mL PYO, or both. While PA14 can produce PYO, the endogenous level of PYO at a low cell density was insufficient to provide sufficient protection. (B) Growth curves of PA14 *Δphz* (*Δphz*) without treatment or treated with 20 μg/mL Strep, 2 μg/mL PYO, or both. All curves were recorded by multi-well plate reader at wavelength of 600 nm. Means ± SD were presented throughout (*n* = 3). The data underlying this figure can be found in S1 Data. Strep, streptomycin; WT, wild type.
(TIF)

S7 Fig Supernatant from a high-density culture of PA14 provided antibiotic tolerance in PAO1. (A) Growth curves of PAO1 treated with 20 μg/mL Strep and with or without the supernatant of PA 14 (WT supernatant). (B) Growth curves of PAO1 in the presence of 20 μg/mL Strep and with or without the supernatant of PA14 *Δphz* (*Δphz* supernatant). All curves were recorded by multi-well plate reader at the wavelength of 600 nm. Means ± SD were presented throughout (*n* = 3). The data underlying this figure can be found in S1 Data. Strep, streptomycin; WT, wild type.
(TIF)

S1 Data Figure source data. (XLSX)

## Abbreviations

A600 nm: absorbance 600 nm
ATCC: American Type Culture Collection
BHI: brain-heart infusion
CLSI: Clinical and Laboratory Standards Institute
ESBL: extended-spectrum beta-lactamase
Kan: kanamycin
LB: Luria-Bertani
MIC: minimum inhibitory concentration
PYO: pyocyanin
QS: quorum sensing
ROS: reactive oxygen species
