# Supplementary figures and images for: Universal antibiotic tolerance arising from antibiotic-triggered accumulation of pyocyanin in Pseudomonas aeruginosa
Source: PLoS Biol. 2019 Dec 16;17(12):e3000573. doi: 10.1371/journal.pbio.3000573 (PMC6936868; doi:10.1371/journal.pbio.3000573)

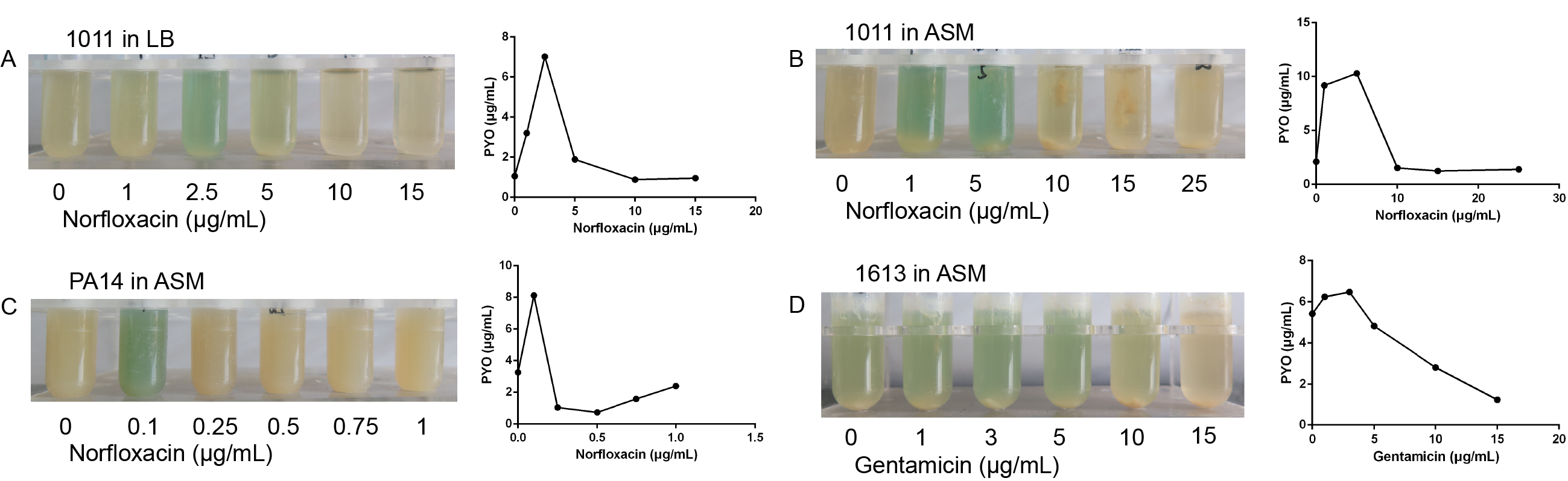

Supplement: S2 Fig — (A) P. aeruginosa 1011 was treated with norfloxacin in LB for 16 h. P. aeruginosa 1011 was treated with norfloxacin in ASM for 20 h. (B) PA14 was treated with norfloxacin in ASM for 20 h. (C) P. aeruginosa 1613 was treated with gentamicin in ASM for 24 h. (D) The clinical P. aeruginosa isolates (strains 1011 and 1613) were kindly gifted by Professor Rong Zhang at The Second Affiliated Hospital of Zhejiang University (Hangzhou, China). ASM was prepared according to the protocol shared by Sriramulu Diraviam Dinesh (Protocol Exchange, 2010; doi:10.1038/protex.2010.212). All of the P. aeruginosa were cultured in tubes with shaking of 200 r/min at 37°C. The data underlying this figure can be found in S1 Data. ASM, artificial sputum medium; MIC, minimum inhibitory concentration. (TIF) [file pbio.3000573.s003.tif]

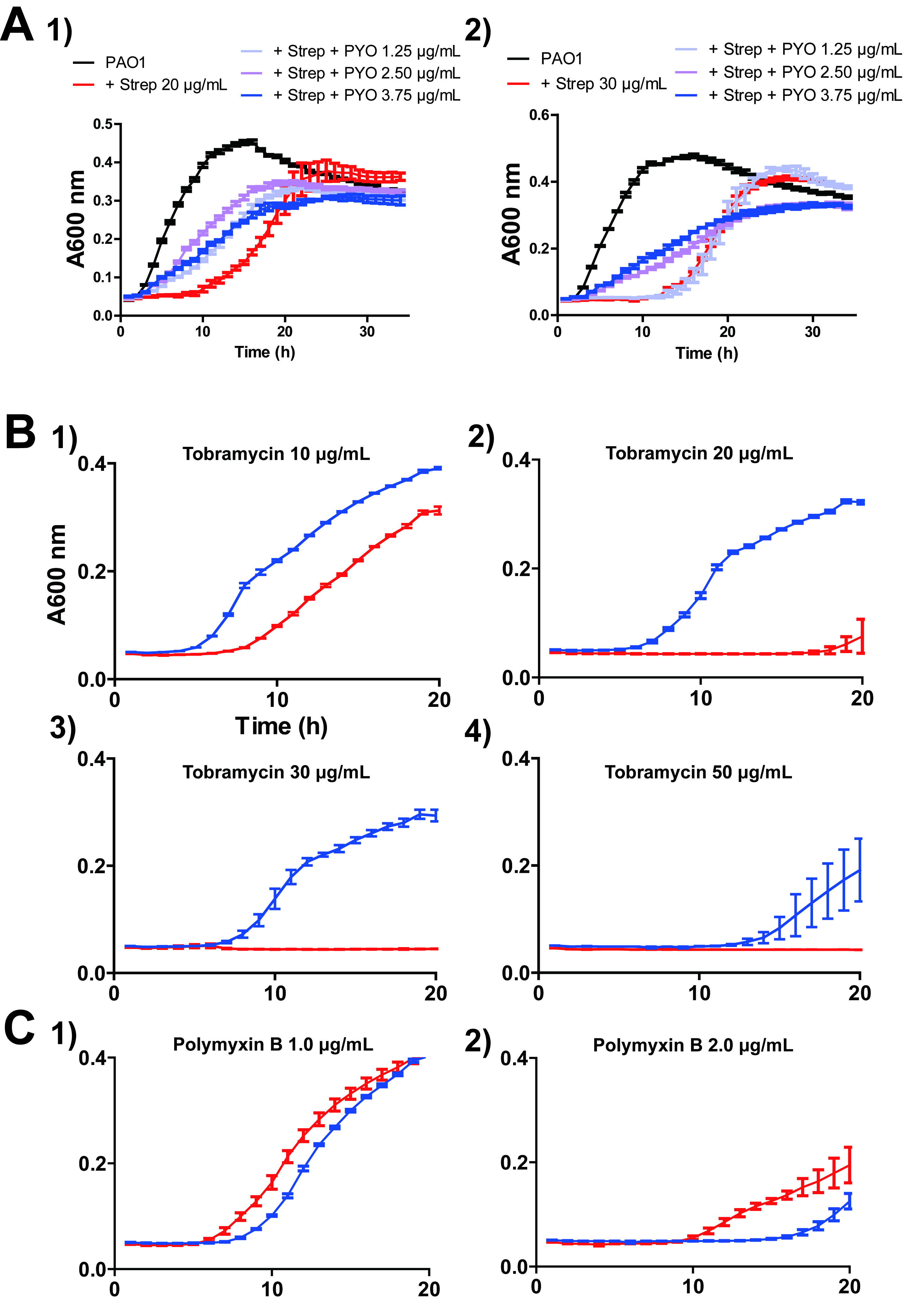

Supplement: S3 Fig — (A) The tolerance was PYO-dose dependent. PAO1 was treated with 20 μg/mL Strep and 30 μg/mL Strep. Culture of P. aeruginosa PAO1 at exponential phase (A600 nm = 0.2) was diluted in LB broth, then treated with different antibiotics, in the presence (blue line) or absence (red line) of 2 μg/mL PYO. (B) Growth curves of PAO1 treated with varying concentrations of tobramycin (from 10 μg/mL to 50 μg/mL, as indicated), in the presence (blue line) or absence (red line) of 2 μg/mL PYO. (C) Growth curves of PAO1 treated with polymycin B (at 1 μg/mL and 2 μg/mL), in the presence (blue line) or absence (red line) of 2 μg/mL PYO. A600 nm was measured by a plate reader. Means ± SD are presented throughout (n = 3). The data underlying this figure can be found in S1 Data. Strep, streptomycin. (TIF) [file pbio.3000573.s004.tif]

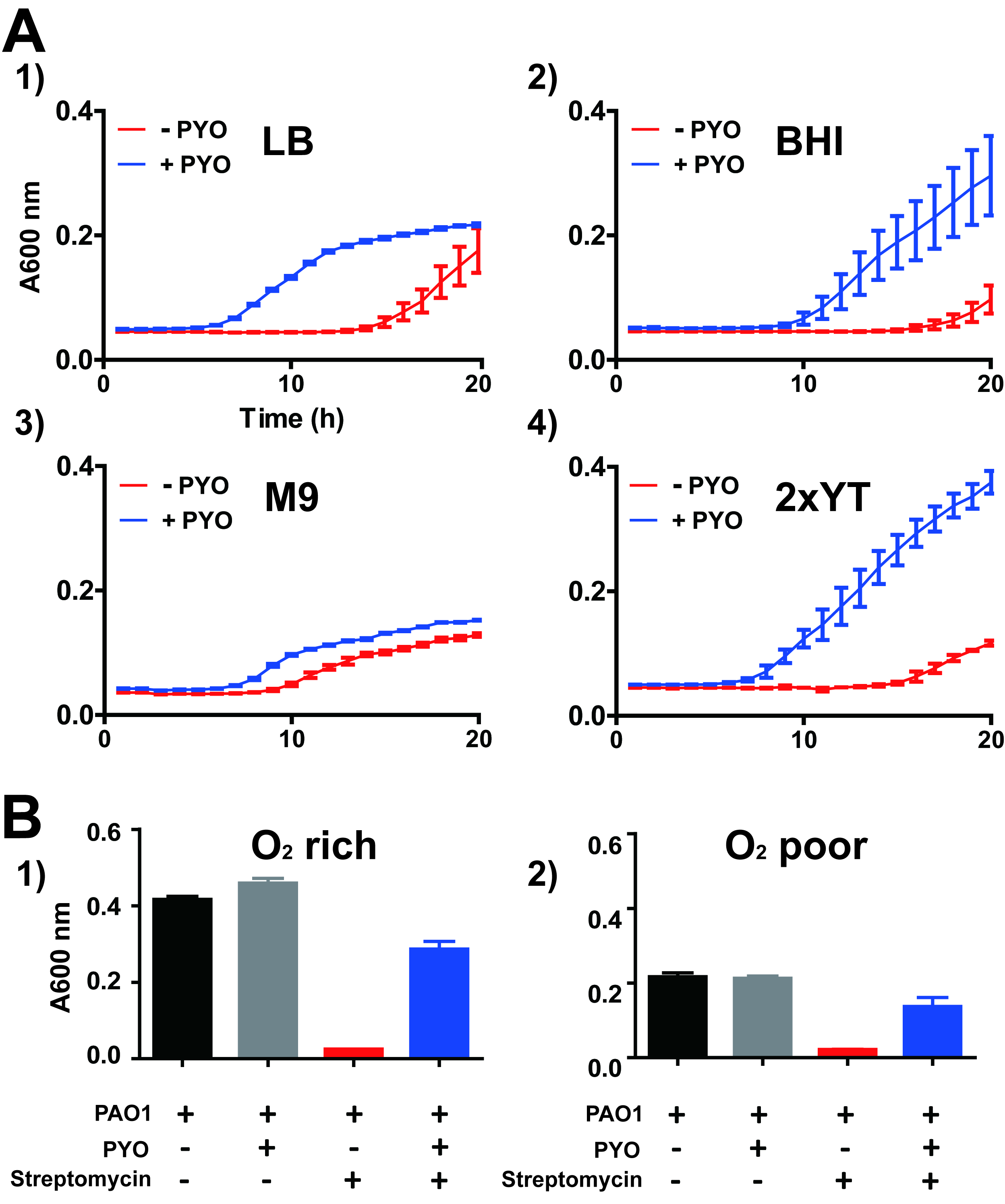

Supplement: S4 Fig — (A) PYO-mediated tolerance in LB broth (A-1), BHI (A-2), M9 (A-3), and 2xYT (A-4) media. Cultures of P. aeruginosa PAO1 were inoculated into the media in the presence of 20 μg/mL streptomycin and 2 μg/mL PYO. The growth curves were measured by A600 nm in a 96-well plate with a plater reader. Means ± SD were presented throughout (n = 3). (B) PYO-mediated tolerance in both oxygen-rich and oxygen-poor environments. P. aeruginosa PAO1 reached higher densities in an oxygen-rich condition (wells were sealed with oxygen-permeable membrane) than in an oxygen-poor condition (wells were covered with mineral oil). The ratios are 0.749 and 0.650 under oxygen-rich and oxygen-poor conditions, respectively. Means ± SD were presented throughout (n = 6). The data underlying this figure can be found in S1 Data. (TIF) [file pbio.3000573.s005.tif]

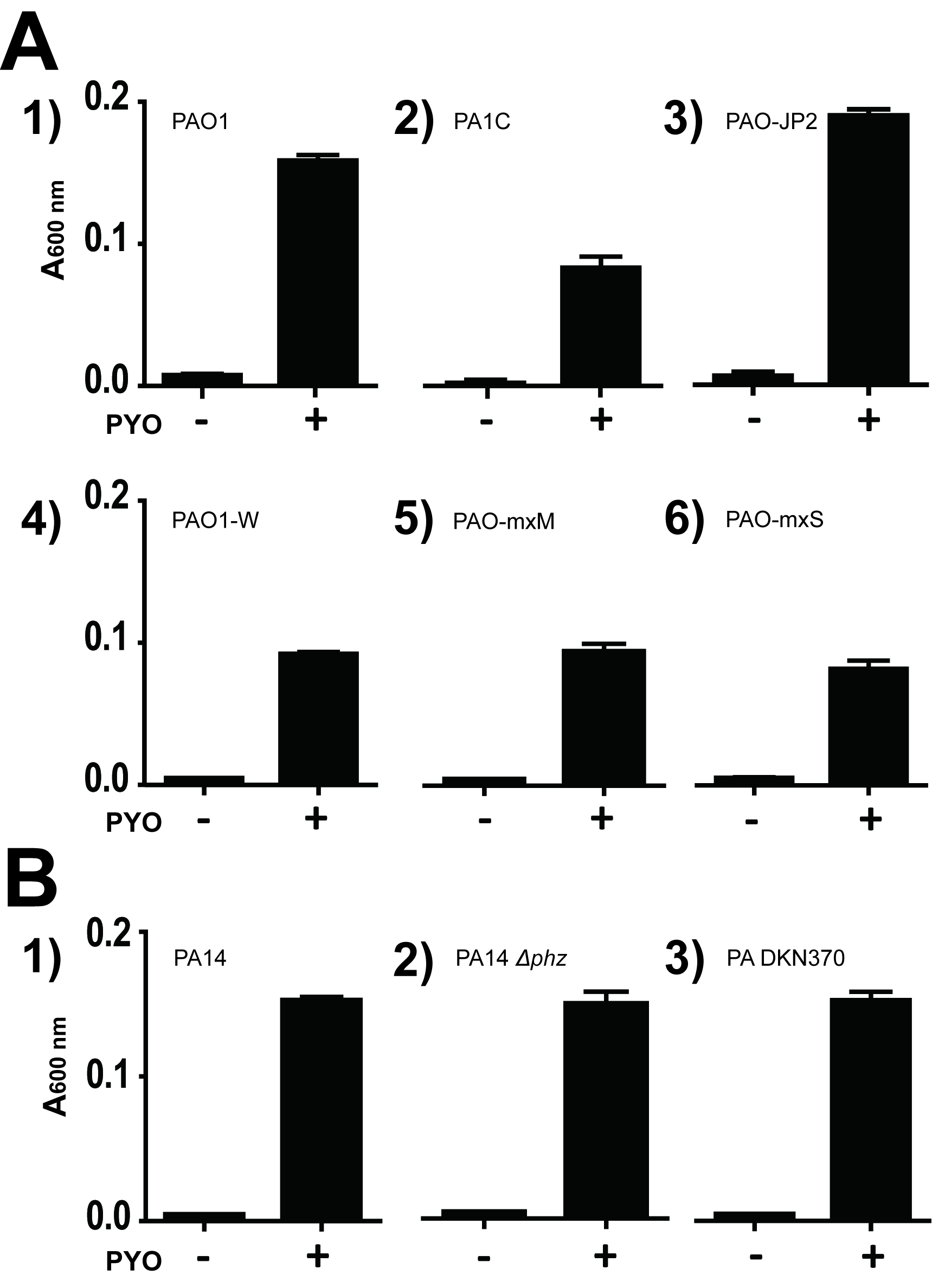

Supplement: S5 Fig — (A) PYO-mediated tolerance in P. aeruginosa PAO1 and mutants. PAO1 (A-1), PA1C (A-2), PAO-JP2 (A-3), PAO1-W, and mutants (A-4 to A-6) were treated with 20 μg/mL, 30 μg/mL, 20 μg/mL, and 30 μg/mL streptomycin, respectively, in the presence or absence of 2 μg/mL PYO. (B) PYO-mediated tolerance was independent of the endogenous PYO production. PA14 (B-1, wild type), PA14 Δphz (B-2, no PYO production), and DKN370 (b-3, PYO-overproducing strain) were treated with 20 μg/mL streptomycin in the presence or absence of 2 μg/mL PYO. All data were plotted at 10 h after inoculation. Means ± SD are presented throughout (n = 6). This apparent independence was likely due to the lack of sufficient PYO accumulation at an initially low-density culture, even if a strain has the ability to produce PYO. Also see results in S6 and S7 Figs. The data underlying this figure can be found in S1 Data. (TIF) [file pbio.3000573.s006.tif]

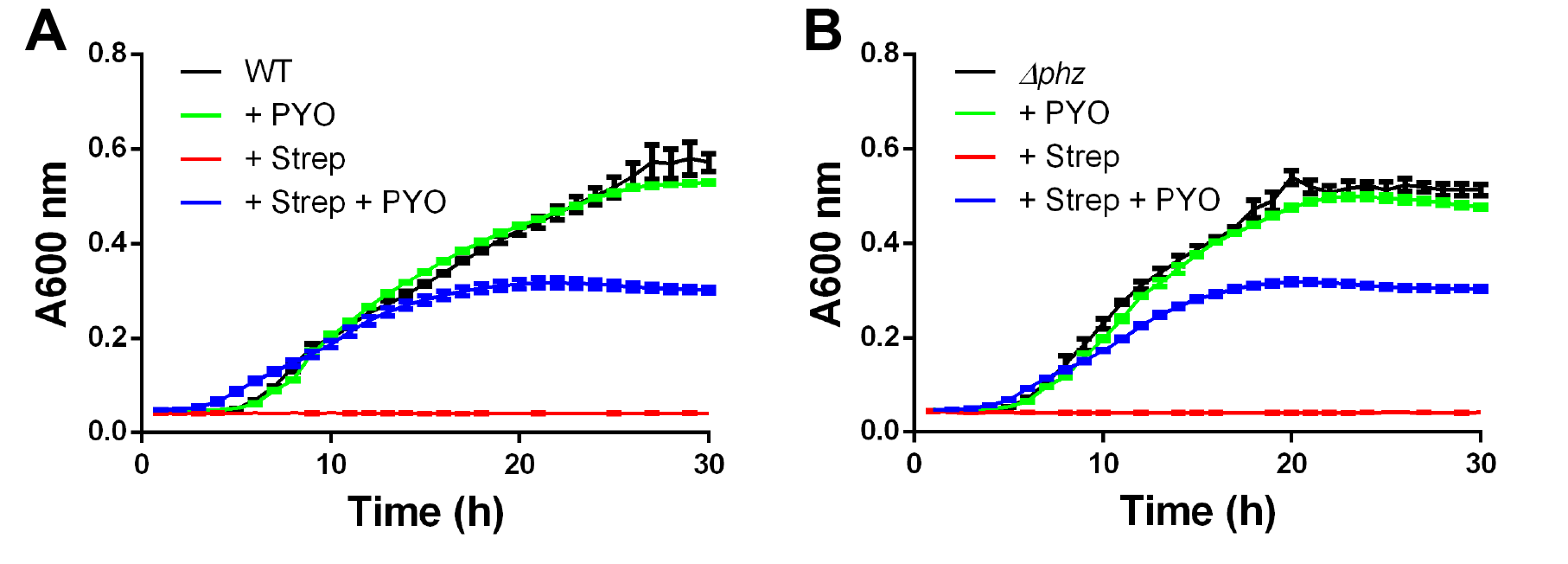

Supplement: S6 Fig — (A) Growth curves of PA14 (WT) without treatment or treated with 20 μg/mL Strep, 2 μg/mL PYO, or both. While PA14 can produce PYO, the endogenous level of PYO at a low cell density was insufficient to provide sufficient protection. (B) Growth curves of PA14 Δphz (Δphz) without treatment or treated with 20 μg/mL Strep, 2 μg/mL PYO, or both. All curves were recorded by multi-well plate reader at wavelength of 600 nm. Means ± SD were presented throughout (n = 3). The data underlying this figure can be found in S1 Data. Strep, streptomycin; WT, wild type. (TIF) [file pbio.3000573.s007.tif]

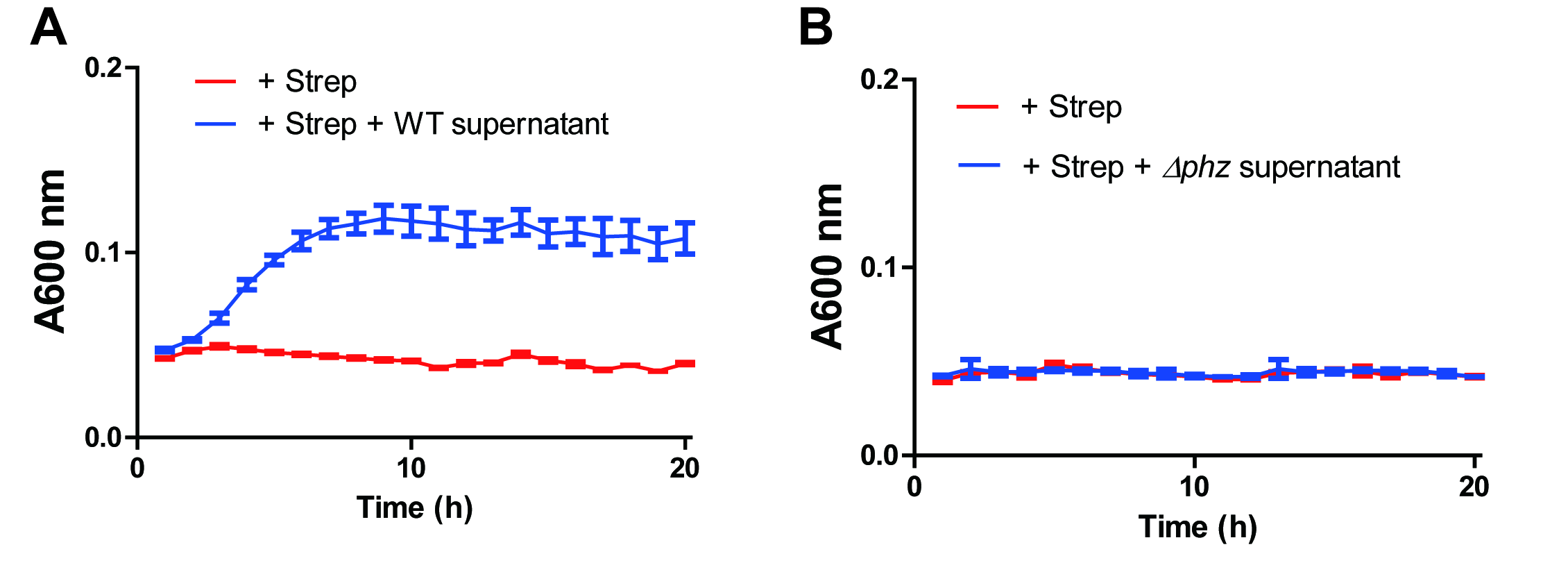

Supplement: S7 Fig — (A) Growth curves of PAO1 treated with 20 μg/mL Strep and with or without the supernatant of PA 14 (WT supernatant). (B) Growth curves of PAO1 in the presence of 20 μg/mL Strep and with or without the supernatant of PA14 Δphz (Δphz supernatant). All curves were recorded by multi-well plate reader at the wavelength of 600 nm. Means ± SD were presented throughout (n = 3). The data underlying this figure can be found in S1 Data. Strep, streptomycin; WT, wild type. (TIF) [file pbio.3000573.s008.tif]
